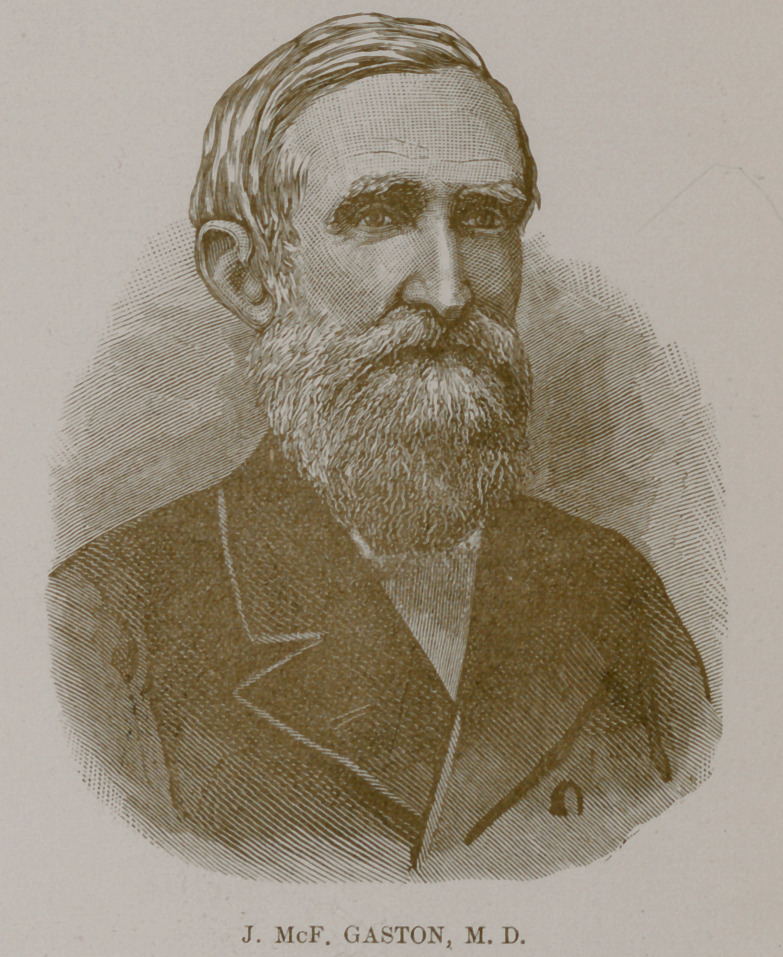# Our Portrait Gallery

**Published:** 1885-03

**Authors:** 


### OUR PORTRAIT GALLERY.

### JAMES MCFADDEN GASTON, M. D.

The subject of this sketch was born in Chester District, South Carolina, December 27, 1824. He comes of the Gaston family of South Carolina who made themselves famous in the Revolutionary war. His great-grandfather was the first to organize armed resistance to the British in Chester District.

Dr. Gaston's early life was spent in the eastern part of Chester District, where he attended the ordinary schools until he was sixteen years old, when he entered the South Carolina College, from which institution he graduated in 1843. He began the study of medicine soon after leaving college, under the preceptorship of his father, Dr. J. B. Gaston, and attended his first course of lectures in 1844–45 at the University of Pennsylvania, and graduated in medicine the following winter in Charleston. Soon after his graduation in medicine Dr. Gaston entered into a partnership with his father and practiced medicine in Chester for six years. At the expiration of this time he was married to Miss Sue G. Brumby, daughter of Prof. Brumby, who then occupied the chair of Chemistry in the South Carolina College at Columbia, which led to his adoption of that city as his future home.

In 1861 Dr. Gaston enlisted in the Columbia Greys as a pri-

J. McF. GASTON, M. D.

vate. Soon after this company entered the service he was appointed Chief Surgeon of the South Carolina forces under the command of Gen. M. L. Bonham, and ordered to duty upon his staff as medical director of the department.

Surgeon Gaston accompanied Gen. Bonham to Richmond, Va., where he remained until the removal of the troops to Manassas, where he was announced in orders as medical director of the department under Gen. G. T. Beauregard.

After the battle of Manassas, where Dr. Gaston served as Medical Director, at his own request he was assigned by Gen. Beauregard to duty with the Third Brigade South Carolina Volunteers, under command of Gen. D. R. Jones.

He remained with the Third Brigade when under the command of Brigadier General R. H. Anderson, and when he was made Major-General he manifested the estimate placed upon Surgeon Gaston by making application to have him assigned as Chief Surgeon of his Division.

Thus he became identified with Anderson's Division and passed through the stirring compaigns in which it bore such an active part in Virginia and Pennsylvania.

While on sick leave with his family in South Carolina upon the occurrence of the battle of Chicamauga he telegraphed the Surgeon General of his readiness to repair to the relief of the wounded, and was so ordered. Here he labored assiduously for several days with the lamented Dugas.

He then went to Marietta and assisted in the secondary operations required, impressing the Medical Director of hospitals, Dr. S. H. Stout, in such a manner that he subsequently applied for the transfer of Surgeon Gaston to his department as inspector of hospitals. With such an understanding an application was sent up requesting to be relieved from field service and to be assigned to hospital service, but instead of receiving orders to report to

Surgeon Stout, the general order relieving him from the duties of Chief Surgeon of Anderson's division, in compliance with his request, was accompanied with an order to report to the Medical Director of Hospitals in General Beauregard's department at Charleston, S. C. He was then ordered to Fort Gaines to establish a general hospital, remaining here only a short time when he was ordered to Fort Valley, Ga., where he established a hospital and remained until the close of the war.

Soon after the close of the war Dr. Gaston determined to seek a foreign land, and accordingly he made his way to New York in his Confederate grey and took passage for Brazil.

After arriving at Rio de Janeiro the Imperial Government of Brazil tendered the position of Consulting Surgeon on the medical staff to Dr. Gaston, with the rank and pay of the highest medical officer in the army. Dr. Gaston had seen and done enough in this line for the past four years, and declined the appointment, as he wanted to explore the country with a view of removing his family there.

For six years after the family of Dr. Gaston went to Brazil he practiced his profession in the interior towns. At the end of this time he went to Rio de Janeiro to undergo the requisite examination and present a thesis for recognition by the faculty of the Imperial Government. After remaining here four months this was accomplished, and early in 1874 he removed to the thriving and populous city of Campainas, in the Province of S. Paulo, where he remained until his removal to the United States at the close of 1883.

Atlanta had been selected as Dr. Gaston's future home before leaving Brazil. Soon after his arrival here he inaugurated a surgical infirmary upon a basis that he had adopted for a similar institution in Brazil.

In his broad field of experience as a general practitioner has been included more than ordinary attention to obstetrics, gynecology and surgery, so that his literary productions cover a variety of subjects such as are rarely treated so thoroughly by one observer. He has likewise claimed a prominent place among neurologists of the present day by his lucid exposition of the excito-dynamic element of the nervous system.

The early literary productions of Dr. Gaston appeared in the *Charleston Medical Journal*, as follows: "Treatment of Typhoid Fever;" "Use of Bozeman's Button Suture in recto-vaginal Fistula;" "Cure of Traumatic Tetanus with Lobelia Inflata;" "The Application of Adhesive Plaster for counter-extension in Fracture of the Femur."

Some contributions of importance were made during that period to the South Carolina Medical Association, of which we note: "The Abdominal Spring Pessary in the Treatment of Uterine Displacements; a Criterion for the use of Stimulants;" "The Correlation of the Nerves and Capillaries with the Internal Organs."

"A report of a unique and successful restoration of the small intestine by the introduction of seven loops of silver wire, after losing 2½ ft. of the canal," was incorporated in the military surgery of Dr. J. J. Chisolm, published at the opening of the civil war.

In the New Orleans *Medical and Surgical Journal* have appeared: "The Binodide of Mercury in the treatment of Syphilis;" "Local Spasm of the Womb with retention of Placenta and Hemorrhage;" "Observation on the Etiology, Pathology and treatment of Rheumatism;" "Leprosy, as seen in Brazil;" "Clinical Lecture on Perforated Fracture of the Cranium, followed by Epilepsy and Operation with the Trephine."

The following papers have been published in the *American Journal of Medical Sciences*: "Dynamoscopy;" "Report on Dr. Laboia's division of the Os Uteri by Ligature;" "Use of Ecraseur for curing deep-seated Fistula in ano."

A series of interesting and elaborate contributions have been published in the *Medical and Surgical Reporter* within a few years, consisting of "The Innovations of Medical and Surgical practice;" "Ablation of the womb with Fibroid Tumor;" "Practical observations on Tetanus;" "The unknown factor in the equation of the Nervous System, or the action of Medicine on the Cure of Disease;" "Successful treatment of psoas Abscess."

A number of important articles have appeared in *Gaillard's Medical Journal*, of which the most important are: "Brazil in its relation to Medicine." "Errors in diagnosis, with report of ligations of femoral and subclavian arteries," Complications in Cases of Twins;" "Ligations of Femoral Artery in Sanguinous Effusion below the popliteal Region;" "A plea for the Pessary;" "Distinguishing features of malignant pustule and anthrax;" "Suppression and Deficiency of Bile, with Atony of Liver;" "Medication through superficial and deep-seated Tissues;" "Obstruction of the Gall-duct and its bad consequences with remedial operation suggested."

His philosophical essay giving an "Explanation of the Pathology and Therapeutics of the Diseases of the Nerve-centres, especially Epilepsy," forms a part of the volume of Transactions of the Medical Association of Georgia for 1884.

The following articles have appeared in the *Southern Medical Record:* "Hypodermic use of Carbolic Acid for the cure of Erysipelas;" "Silver wire sutures in Wounds of the Intestines;" "Obstruction of the ureter, its consequences and remedy;" "Remarks on taking the chair of surgery in the Southern Medical College;" "Laparotomy with suture of Intestinal Canal;" "Medical and Surgical uses of Spirits Turpentine."

In the *Atlanta Medical Register* "The Outlook of Medicine and Surgery" occupied a prominent place during the year 1883.

The first article in the new series of this JOURNAL was by Dr.

Gaston, and he has been a constant contributor up to the present time, his valuable papers having appeared in the following order: "Extreme Anæsthesia for obstetric Operations;" "Division of Stricture without subsequent sounding;" "Public Health," "Experimental Cholecystotomy;" "Review of elementary Principles of Electro-Therapeutics;" "Clinical Lecture on Dropsy, complicated with Aneurism;" "Anomalous Tumor;" "Report of antepartum hour-glass Contraction;" "Review of Transactions of the Medical Society of the State of Pennsylvania."

Besides these, Dr. Gaston has published an interesting book of four hundred pages, entitled "Hunting a Home in Brazil." Other lesser articles upon medical and surgical topics have been published in various periodicals, and while in Brazil some contributions were made to the journals in the Portuguese language among which is a translation of the able and practical article of Dr. R. H. Day upon the treatment of yellow fever, while we have before us a pamphlet from his pen entitled "Allopathics and Homœopathics," and his printed thesis presented to the *Echole de Medicina de Rio de Janeiro* on the subject of "Electro-therapia."

The election of Dr. Gaston to the Chair of Surgery in the Southern Medical College was an evidence of the estimate placed upon his professional attainments.

The general appreciation of the character and bearing of Dr. Gaston by his colleagues, here and elsewhere, is the highest tribute to his worth which could be received by a professional man.

The original investigations, made by him since making his home among us, have satisfied the members of the medical profession everywhere that he is preëminently practical and judicious in all his undertakings.

## Figures and Tables

**Figure f1:**